# Analytic Advances in Social Networks and Health in the Twenty-First Century

**DOI:** 10.1177/00221465221086532

**Published:** 2022-04-08

**Authors:** Alexander Chapman, Ashton M. Verdery, James Moody

**Affiliations:** 1 The Pennsylvania State University, University Park, PA, USA; 2 Duke University, Durham, NC, USA

**Keywords:** health, social networks, text analysis

## Abstract

The study of social networks is increasingly central to health research for medical sociologists and scholars in other fields. Here, we review the innovations in theory, substance, data collection, and methodology that have propelled the study of social networks and health from a niche subfield to the center of larger sociological and scientific debates. In particular, we contextualize the broader history of network analysis and its connections to health research, concentrating on work beginning in the late 1990s, much of it in this journal. Using bibliometric and network visualization approaches, we examine the subfield’s evolution over this period in terms of topics, trends, key debates, and core insights. We conclude by reflecting on persistent challenges and areas of innovation shaping the study of social networks and health and its intersection with medical sociology in the coming years.

Scholars increasingly recognize that interactions, personal relationships, and social networks are crucial to understanding all manner of health phenomena. However, little research simultaneously studied social networks and health until late in the twentieth century despite long traditions formally studying either social networks (Moreno 1934; Simmel 1902)^1^ or health (Graunt 1662; Snow 1855). During the 1990s and early 2000s, stimulated in part by funding and interest in the HIV/AIDS epidemic, sociologists led several key innovations at the intersection of research on social networks and health, building a new scholarly paradigm.

Since 2000, twice as large a proportion of health articles in academic journals reference networks, and the number of articles addressing both social networks and health has sextupled, growing quicker than general academic or sociological publishing over the period. Likewise, the total value of grants and contracts awarded to network studies by the National Science Foundation (NSF) and National Institutes of Health has increased tenfold, also faster than total awards or sociological awards (Figure 1). Prior to 1990, empirical studies of networks primarily addressed topics in organizational sociology (e.g., Granovetter 1973), demography (e.g., Bott 1955), community organization (e.g., Fischer 1982; Wellman 1979), and diffusion of innovations (Rogers 1962). Although there are notable exceptions (e.g., Berkman and Syme 1979; Cobb 1976; Kandel 1975), few network studies addressed health. However, early innovations in social network research, including methodological works (e.g., Frank and Strauss 1986), brought new and growing attention to the topic. For example, of the 58,000 articles that cite Granovetter’s (1973) work on the “strength of weak ties,” more than 50,000 are after 2000 (based on our analysis of Google Scholar data^2^).

**Figure 1.**
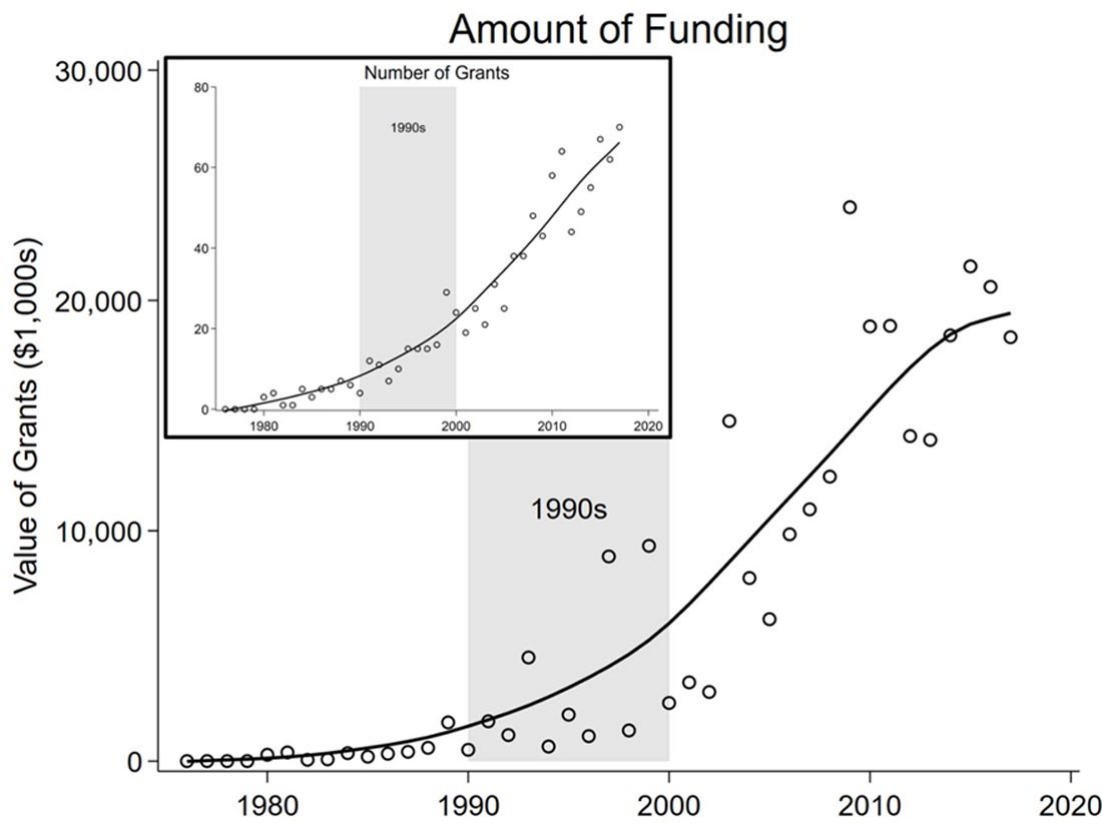
Value and Number of NSF Grants at Sociology-Relevant Divisions with “Network Analysis” in the Title. *Note*: NSF awarded the following numbers of grants over the plotted time frame: 8,139 (1980), 9,744 (1990), 10,365 (2000), and 13,601 (2010). Likewise, the NSF’s Directorate for Social, Behavioral and Economic Sciences awarded 540 (1980), 650 (1990), 979 (2000), and 1,326 (2010) over this time frame. Thus, the growth in social networks–related grants outpaces changes in numbers of grants NSF awarded overall and in its most relevant division. NSF = National Science Foundation.

Our thesis is that sociologists were essential to the turning point in social networks and health scholarship in the 1990s and early 2000s. We base this argument on close reading and bibliometric and network visualization analyses of publication, citation, and funding data from numerous sources that allow us to examine the condition of social networks and health research before the late 1900s and its evolution since. We review innovations in theory, substance, data, measurement, and modeling that made such advances possible. The expansion of social networks and health research continues unabated because there is growing interest in and ability to study social networks and health. Furthermore, once-in-a-century health crises such as coronavirus disease-2019 (COVID-19) demand additional attention to the relationships between social networks and health, most directly in terms of disease diffusion, the diffusion of (and opposition to) disease-mitigation behaviors and policies (e.g., masking and vaccinations), and the effects of social isolation on any number of health outcomes (Pfefferbaum and North 2020). The current period is timely for reflecting on developments during the last three decades and highlighting current challenges and opportunities. Such an effort clarifies where medical sociologists could contribute new insights to the broader scientific community and where they could look to import some ideas.

## Social Networks and Health: Pre-1990

Fifty years ago, little scholarship linked social networks and health (Berkman et al. 2000), which is surprising because early developments in social network analysis concentrated on mental health (Loomis 1941; Moreno 1934), and important midcentury publications deeply connected the concepts (Festinger 1954; Makowsky 1969; Rogers 1962). Much of this work was sociological, but social networks and health scholarship had yet to achieve a broad and lasting purchase in the health sciences more generally.

### Preludes to Integrating Social Networks and Health

The 1970s were a key launching point for the widespread use of social network approaches, marked by institution building (e.g., founding of the International Network for Social Network Analysis in 1977), theory expansion (e.g., Granovetter 1973; Wellman 1979), conceptual advances regarding the distinction between selection and influence (Kandel 1975), and breakthrough theories pointing to networks and health-linking mechanisms (e.g., Berkman and Syme 1979; Cobb 1976). Despite this activity, the volume of research on social networks and health was scant compared to today. Web of Science and Google Scholar data suggest there were only 16 articles published on both social networks and health between 1970 and 1979.^3^ The influence of these article is substantial but grew slowly at first and surged in subsequent decades. Take, for example, Sidney Cobb’s (1976) Presidential Address to the American Psychosomatic Society about how social support moderates stress. The article has, as of our writing, 10,500 citations according to Google Scholar, and, as a measure of its secondary impact, more than 100 of its citing articles have 1,000 citations or more (e.g., several prominent works by sociologists James House and Debra Umberson [House et al. 1985; House, Landis, and Umberson 1988; House, Umberson, and Landis 1988; Umberson 1987] and Cohen and Wills’s [1985] social buffering hypothesis, which itself has over 18,000 citations). However, this impact was slow to build. Only 115 articles cited Cobb’s (1976) address in the 1970s; 1,610 cited it in the 1980s, 1,460 in the 1990s, 2,200 in the 2000s, and over 5,000 have cited it in the past decade.

The 1980s were also a time of foundational social networks scholarship that produced important contributions to network theory (e.g., Coleman 1988; Wellman and Wortley 1990) and methods (e.g., Frank and Strauss 1986; Holland and Leinhardt 1981; Wasserman 1980). Sociologists made many of these contributions, paving the way for the development of a literature focused on social networks and health. Sociologist James Coleman’s (1988) connection between social capital and education (thereby connecting to health) remains highly relevant despite being more than 30 years old; it garnered more than 2,800 citations in 2019 alone. Perhaps in reaction to the person-centered rational choice models popular in the 1980s, Bernice Pescosolido, a medical sociologist, led a series of pivotal studies that situated (mental) health choices in social network contexts (Pescosolido 1986; Pescosolido and Georgianna 1989). These efforts are still bearing fruit within research on mental health (Perry and Pescosolido 2012, 2015).

Likewise, the advances in social network analysis techniques of the 1980s would become central components of core network methodologies used in a variety of fields, including the health sciences. Perhaps the clearest of these connections can be traced from work on Markov random graph models (Frank and Strauss 1986), which overcame assumptions of case independence, through p* models (Anderson, Wasserman, and Crouch 1999) to what is today known as exponential random graph models (ERGMs). ERGMs enable modeling structural network influences on tie creation, like how friends of friends tend to become friends, while accounting for interdependence concerns (Robins et al. 2007; Snijders 2002; Snijders et al. 2006). These advances and associated tools (Hunter et al. 2008), many authored by sociologists (e.g., Carter Butts, Mark Handcock, Tom Snijders), are widely used in the study of health.

Despite these advances in the 1980s, there remained few studies explicitly at the intersection of networks and health at this time. Scholars published 66 articles in the 1980s on social networks and health, nearly two-thirds of them in 1985 to 1989. Major sources of external funding for social networks research remained limited, and methodological challenges and a lack of social network data posed substantial impediments. For instance, the NSF sponsored only 12 grants on the topic of “network analysis” in the 1980s, averaging $82,000 per grant, and 5 of these 12 were funded in 1988 or 1989.

## The Dam Breaks: Key Moments of the 1990s and Early 2000s

Three key facilitators spurred rapid development of social networks and health research in the 1990s: the HIV epidemic, research on adolescent networks, and advances in software tools and statistical models that account for the dependent nature of network data.

### Role of the HIV Epidemic

The deluge of research in social networks and health in the 1990s coincided with rapid research and funding expansion associated with the HIV/AIDS epidemic. At the cusp of the 1990s, there were 100,000 cases of HIV/AIDS reported in the United States, a grim milestone since the country’s first reported case in 1981 and a sizeable fraction of the 8 to 10 million worldwide total (Chin 1990). In the early 1990s, knowledge of numerous celebrities combating HIV became widespread, furthering public attention. In 1990, the U.S. federal government awarded just over $2 billion to HIV/AIDS grants and contracts, with funding ballooning to $11 billion by 1999 (Kaiser Family Foundation 2019). This funding expansion catalyzed new scholarship on sexual and needle-sharing contacts, key HIV transmission modalities.

The HIV epidemic demanded network theory development because common epidemiological techniques tuned to easy-to-pass infections like influenza were ineffective for explaining HIV’s spread. This led to a detailed examination of network spread processes, reviving early sociological models (Rogers and Kincaid 1981). The question was how do networks channel and facilitate the spread of hard-to-pass infections. Building on earlier, detailed studies of sexual networks (Klovdahl 1985; Potterat 2015; Rothenberg 1983), sociologist Martina Morris (1993) keyed off the expansions, bridging network and epidemiological models. The dynamics of the HIV/AIDS epidemic never fully matched the traditional mathematical models. Consequently, Morris and colleagues examined partner–timing dynamics (Kretzschmar and Morris 1996; Morris and Kretzschmar 1997), ushering in theoretical and methodological contributions on the diffusion limits due to relational timing, especially “concurrent” sexual relations, relations that overlap in time. Using simulation studies, they showed that higher concurrency rates hasten disease spread, insights later confirmed with empirical data (Aral et al. 2004; Helleringer et al. 2014).

The HIV epidemic magnified the need for empirical data on risk networks, both domestically and internationally, such as the work led by epidemiologists and sociologists like Richard Rothenberg, Jonn Potterat, and Susan C. Watkins. In the United States, this push for new data included multiple small-scale studies of HIV risk groups. Perhaps the most impactful was “Project 90” (Klovdahl et al. 1994), which attempted to trace social, drug, and sexual contacts of every “high risk” actor in downtown Colorado Springs over multiple years (see Potterat 2015). Importantly, the study focused on multiple types of ties, or multiplex ties, such as sexual contact and co-injecting. Project 90 showcased how complete network data reveal chains of connections indirectly linking not directly tied actors (i.e., global, saturated, or “sociocentric” network data) and allowed new understandings of disease dynamics. The 1990s HIV epidemic across Africa and Southeast Asia also jumpstarted international network data collection, sociocentric and “egocentric” (i.e., those that examine only a sample of network actors and their direct ties), including efforts by Martina Morris (Morris et al. 1996; Morris and Kretzschmar 2000), Susan C. Watkins (Bracher, Watkins, and Santow 2003), Carl Latkin (Latkin 1998), and others.

The urgency of the HIV epidemic (and the funding resources invested) also brought networks to the attention of physical scientists, who provided high-profile statements on patterns of disease spread. For example, sociologist Duncan Watts and mathematician Stephen Strogatz (1998) built a model of clustered networks weakly connected by shortcut ties that had significant implications for epidemic spread (because even highly clustered networks can have short distances between all pairs). Somewhat controversially, Barabási and Albert (1999) argued that a “ubiquitous” feature of networks—a skewed distribution of number of partners—signaled an underlying network topology that makes endemic diffusion unstoppable, assertions that have been challenged (Jones and Handcock 2003). These two articles alone—cited 44,000 and 36,000 times, respectively (according to Google Scholar)—opened a floodgate of formal mathematical and computational science contributions to network understandings of spreading processes (e.g., Newman 2003).

Beyond diffusion of the disease, researchers also found that social structure plays a role in HIV transmission through social influence mechanisms (Latkin 1998). Social influence has two meanings: the concept that network proximity channels influence (Marsden and Friedkin 1993) and that norms and comparisons channel influence, possibly indirectly (Landrine et al. 1994). With respect to HIV dynamics, models demonstrated strong patterns of social influence on condom use (DiClemente 1991; Wulfert and Wan 1993). The development of social influence models of health behaviors, although underappreciated at the time (Berkman et al. 2000), directed research into how social influence operates through social networks.

At the same time, disconnected from the HIV epidemic, the 1990s witnessed an explosion in studies on community and social structure. Building on decades of work (Wellman 1979, 1988; Wellman and Leighton 1979), sociologist Barry Wellman authored a fundamental article testing the role of networks in different aspects of social support (Wellman and Wortley 1990). Likewise, there were numerous advances in theoretical models of social capital around this time (Bourdieu and Wacquant 1992; Coleman 1988; Lin 1999, 2002; Portes 1998), culminating in Putnam’s (2000) influential arguments about the importance of and potential decline in social capital.^4^ Sociologist Ichiro Kawachi’s research found attention to social capital is among the most influential sociological exports to public health (Kawachi 2010; Moore and Kawachi 2017).

### Adolescent Health

One result of early concerns over the HIV epidemic was funding for the Add Health study,^5^ a landmark investigation of adolescent sexual risk behavior. Developed by a team of sociologists (including J. Richard Udry, Kathleen Mullan-Harris, and Peter Bearman), Add Health collected a first wave of data from a nationally representative sample of students in scores of middle and high schools across the United States in 1994 to 1995 and has thus far followed this cohort over five waves (1994–1995, 1996, 2001–2002, 2008, and 2016–2018). The availability of a large-scale, representative survey with substantial data on the social connections between people and, crucially, a vast assortment of measures of health behaviors, statuses, and outcomes across diverse contexts provided ample fodder for growing recognition that health and networks are intertwined. Some of the most influential works on social networks and health stem from analyses using Add Health. For instance, “Chains of Affection” (Bearman, Moody, and Stovel 2004) has garnered nearly 1,000 citations per Google Scholar, with about 20 of those works being cited 1,000 times or more.

### Computational Tools and Resources

The 1990s and early 2000s saw a rapid rise in reference texts (e.g., Wasserman and Faust 1994) and computational tools and resources that made social network data easier to find, collect, and analyze. The Internet became publicly available in 1991 and broadly changed academic research, but it uniquely influenced social networks and health research. In addition to its facilitation of computation and collaboration, the organization of the Internet became a central idea in the development of network theories (Bandura 2001; Wellman 1997). Outside the academic sphere, the Internet became a new place of community where network ties, from friendship to marriage, were formed (Rainie and Wellman 2014). Likewise, the Internet made it possible to analyze new, historically disorganized and nondigitized sources, revolutionizing the types of data available for network analysis (Watts 1999).

The development of respondent-driven sampling (RDS) by sociologist Douglas Heckathorn (1997) was another 1990s-era data and methods innovation for social networks and health. RDS is a network sampling protocol that enables network-based sampling of hard-to-survey populations and provides a statistical framework for making sample-to-population inferences (Heckathorn 1997). In RDS, respondents drive peer recruitment, but researchers anonymously track recruitment patterns, which enables statistical inference (Heckathorn 1997; Verdery et al. 2015). RDS achieved substantial purchase in studying populations at high risk of HIV in the early 2000s, cementing its popularity.

The growth of computational resources and advent of new tools went hand in hand with growth in social networks and health research broadly, creating a positive feedback cycle as tools enabled research, which prompted greater user demand for good tools. This decade witnessed jumps in computational power for stand-alone network analysis software like UCINET (Borgatti, Everett, and Freeman 2002)—developed by sociologists Lin Freeman, Bruce MacEvoy, and Steve Borgatti in the 1980s—and new software for large-scale network visualization and analysis such as PAJEK (Batagelj and Mrvar 1998). As these approaches developed, so too did the R programming language, created in 1993, which came to include extensive tools for networks in Statnet (Krivitsky et al. 2003) and igraph (Csardi 2020).

Improvements in computational power coincided with new statistical analysis methods, particularly the consolidation of research around p* models, which advanced the theoretical underpinnings for statistical models of networks (Anderson et al. 1999). Although there were many contributors through the 1990s, much of that research was led by sociologist and statistician Stanley Wasserman (Anderson et al. 1999; Wasserman and Pattison 1996). Such work enabled researchers to estimate network models with logistic regression software and standardized and simplified interpretations, improving accessibility. Later, as weaknesses of 1990s modeling techniques became clear (see Handcock et al. 2003; Snijders 2002), methodologists improved ERGMs by including new specifications that corrected earlier, oversimplified specifications (Robins et al. 2007; Snijders et al. 2006).

## A New World: Social Networks and Health Since the 2000s

**Figure 2.**
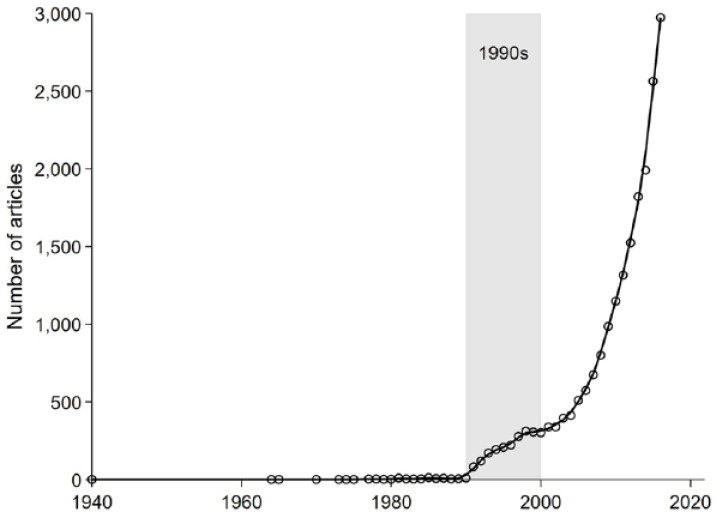
Trends in the Number of Published Articles on Social Networks and Health Since 1940. *Note*: This figure plots the number of English language articles indexed in Web of Science Social Science Citation Index that contain the words “network” and either “health,” “well-being,” or “medicine” in their abstract, title, or journal- or author-identified keywords. There have been 18,572 such articles since 2000.

Research on social networks and health has skyrocketed since these floodgates opened. The share of sociological publications on networks and health after 2000 is more than 5 times greater than that from 1975 to 2000 (based on analysis of Web of Science data^6^). Figure 2 illustrates this point for social science overall (for details, see “Appendix A: Analyses Details for Figure 2 Social Science Publications” in the online version of the journal). There were only one or two publications per year from 1940 until the 1990s, at which point publications began to surge but leveled off by the end of that decade. However, in the early 2000s, an exponential upward trend emerged. There were 1,173 indexed articles in 2013 alone, and the trend shows no signs of weakening: 2,500 articles were indexed in 2019, more than double that of 2013.

**Figure 3.**
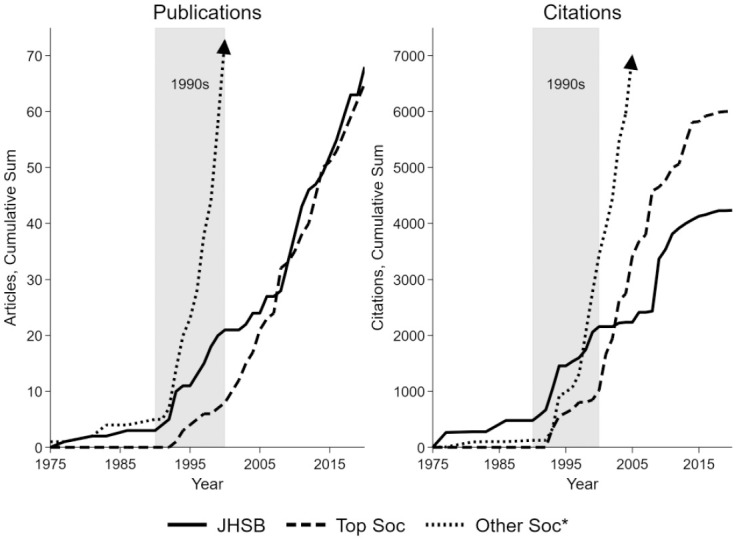
Trends in Number of Publications and Citations to Articles Published in Each Year by Three Categories of Sociology Journals. *Note*: “Other Soc*” capped at year 2000 value in publications and at year 2005 value in citations to maintain scale. This figure shows trends in the cumulative number of articles published on social networks and health articles (left panel) and citations to social networks and health articles published in given years according to Web of Science for articles published in three categories of sociology journals: *Journal of Health and Social Behavior* (*JHSB*); *American Journal of Sociology*, *American Sociological Review*, or *Social Forces* (Top Soc); or all other journals Web of Science indexes in its sociology category (Other Soc). The left panel counts articles, where the cumulative sum increases each year by the number of articles published on social networks and health in that journal in that year. The right panel shows the cumulative sum of citations to articles published in each of the journal categories as of 2020 (e.g., by 2020, there had been just over 4,000 citations to social networks and health articles published in *JHSB*).

This massive growth in research on networks and health was preceded by prominent work in the *Journal of Health and Social Behavior* (*JHSB*). Figure 3 demonstrates this finding by considering cumulative trends in publications (left panel) and citations (right panel) in three journal groups: (1) *JHSB* alone, (2) the “big three” general interest sociology journals (“top soc”: *American Journal of Sociology*, *American Sociological Review*, and *Social Forces*), and (3) all other sociology publications (“other soc,” 225 total outlets; for details, see “Appendix A: Analyses Details for Figure 3 Publication Volume and Citations” in the online version of the article). Prior to the early 1990s, there were about 10 total publications on social networks and health in *JHSB* and about 10 total in the rest of sociology combined, all of them in the 225-journal other soc category and none in the big three (publications panel). Then things exploded in all three groups. With respect to publication impact (citations panel), *JHSB* articles had outsized influence early on and even still, despite being just one outlet, with more than 200 published on the topic. In the Supplemental Material in the online version of the article, we demonstrate that *JHSB*’s early citation influence on social networks and health research owes to contemporaneous citations and not rediscovery of classic work.

### Theoretical and Substantive Advances

Work Since the 2000s further advanced all the developments introduced in the foundational period for social networks and health scholarship. On the substantive side, research has moved from a core focus on biological contagion of HIV to general contagion of health behaviors and outcomes (Centola and Macy 2007), which has necessitated a deep discussion of causal identification in network influence models (Shalizi and Thomas 2011) and growing attention to manipulating network diffusion processes for health interventions (Valente 2012). The availability of network data has exploded, including multiple dynamic data sources, which has itself led to multiple different theoretical and methodological advances.

### Spread of Health Behaviors and Conditions

Arguably, the most influential social networks and health work after 2000 is the work of sociologist Nicholas Christakis and coauthors based on the Framingham Heart Study. These data are notable because the original study was never intended to be a network study, but they were discovered as network data in the 2000s. These authors recognized that the survey-tracking information—the people listed as close contacts to facilitate follow-up—combined with the high sampling rate meant that many named contacts were survey participants. This allowed for a unique, long-term dynamic social network and thus the ability to trace network autocorrelation for multiple health outcomes. The blockbuster article from this work, cited nearly 6,000 times to date (Christakis and Fowler 2007), found a strong correlation between network members’ body mass indices (BMIs). The team has produced similar results across a host of health outcomes, including smoking, depression, happiness, and loneliness (Christakis and Fowler 2013). This line of work posits that behavioral health is contagious, just like HIV or other infectious diseases, a notion whose implicit causal claims remain controversial, reinvigorating attention on causal identification challenges in observational network studies (Christakis and Fowler 2013; Cohen-Cole and Fletcher 2008; Shalizi and Thomas 2011). Despite the controversy, the work has been highly influential.

The problem is that networks and behavior are generally endogenous—although it is possible that health behaviors might transmit between people, people pick friends and close contacts based on their (perhaps unobserved) behaviors. This so-called “selection or influence” argument is pervasive, having been recognized early in the field (e.g., Kandel 1975). This debate is also similar to the nature-nurture debates of genetic sources of health behavior (Caspi 2002), with much the same result: Most careful attempts to disentangle the two (see methods discussion in the following) show that almost all health behaviors result from both selection and influence.

While critics fret over causal identification, health advocates and medical researchers have flipped the script and begun using networks actively for health interventions. While there are multiple types of network interventions (Valente 2012, 2017), perhaps the most common interventions attempt to leverage peer leaders to spread positive behaviors or impede the spread of negative ones (Kim et al. 2015; Osgood et al. 2013). Identified key network players might maximize the spread of trusted information (positive intervention; Kempe, Kleinberg, and Tardos 2015), or they might reduce the spread of unwanted behavior such as smoking (negative intervention; Osgood et al. 2013). Thus far, large-scale randomized trials, such as the Sources of Strength trial that trained peer leaders in suicide prevention (e.g., Wyman et al. 2010) or other network intervention trials to stop those recently infected from spreading HIV (e.g., Nikolopoulos et al. 2016), show promise for both positive and negative network interventions.

### Tools and Techniques

While peer influence and diffusion have shaped recent substantive developments, the ability to statistically model network formation—rather than merely describe network metrics—has been the primary methodological focus since 2000. The 1990s shift to p* models of networks moved the field from descriptive toward inferential network modeling. This shift has been substantively fertile because it enabled testing core theories of network formation, such as the relative importance of preferring to form relations with people like oneself (“homophily”) versus other network processes (“friends of friends become friends”; Goodreau, Kitts, and Morris 2009). These trends also connected network analysis to wider issues in Bayesian statistics (e.g., Hoff, Raftery, and Handcock 2002), a growing area of sociological attention (Lynch and Bartlett 2019).

Prominent new modeling approaches aim to solve the selection or influence endogeneity problem via three distinct approaches. One approach uses instrumental variable models for networks. The basic idea is if social relations can be predicted with a valid instrument (a variable related to the endogenous predictor of interest but, except through that variable, unrelated to the outcome of interest), causal identification is possible (Bollen 2012). While such methods are well known in econometrics, valid instruments are rare. Nonetheless, modern data sources have opened the door for creative efforts. For example, Aral and Nicolaides (2017) used online fitness trackers to test for peer influence on exercise behavior. Because the sample included people from all over the country, they used the weather in a geographically distant peer’s region as an instrument for peer influence (e.g., testing whether those in Phoenix with peers in New York ran more or less depending on peer running behavior predicted by weather patterns in New York).

The second approach, stochastic actor-oriented models (SAOMs), simulate longitudinal network data as a set of balanced utilities within actors, where actors face changing their behavior or their ties, and then uses the resulting best-fit parameters to assess the relative contribution of selection or influence. SAOMs, implemented most accessibly in the Siena software package, claim to sidestep the causal identification problem (conditional on model specification). These models are computationally intensive, but they provide regression-like results to assess peer influence, which increases their relevance to sociologists. Example applications include tests of peer influence related to substance use (McMillan, Felmlee, and Osgood 2018) and links between networks and depression (Schaefer, Kornienko, and Fox 2011). Such work is growing in influence: Schaefer et al. (2011) has more than 200 citations (according to Google Scholar data). The final approach is to focus on sensitivity analyses of sundry sorts (cf. VanderWeele 2011). These models recast the endogeneity problem fundamentally as one of omitted confounders and allow one to assess the robustness of observed relations, which is especially useful when the data will not allow other approaches.

### Network Data Are Everywhere

Since 2000, scholars have begun multiple new networks-focused data collections (e.g., PROSPER), including integrated biomarkers, genes, and other individual-level physical measures to existing social network data (e.g., Panel Study of Income Dynamics, Add Health), and found new ways to collect vast amounts of social network data because of Internet access and software that enable web-scraping and data mining.

New data sources like PROSPER and others collected in the Netherlands, Germany, Honduras, and elsewhere expanded the number of studies that contained consistently measured, sociocentric network data across multiple contexts (Perkins, Subramanian, and Christakis 2015), which enabled hierarchical linear modeling approaches to understand contextual differences (Kim et al. 2015; Lubbers and Snijders 2007). Adding genetic information to existing data sources allowed researchers to detangle the effects of genes, environments (including social ties), and their interactions (Boardman, Domingue, and Fletcher 2012; Fowler, Dawes, and Christakis 2009). Social media and dating websites offered researchers access to relational ties and other relevant health information (Lewis 2013), unveiling research agendas in online romance (Lewis 2013), cyber victimization (Felmlee and Faris 2016), and other health-related consequences of online interactions.

## State of the Field

**Figure 4.**
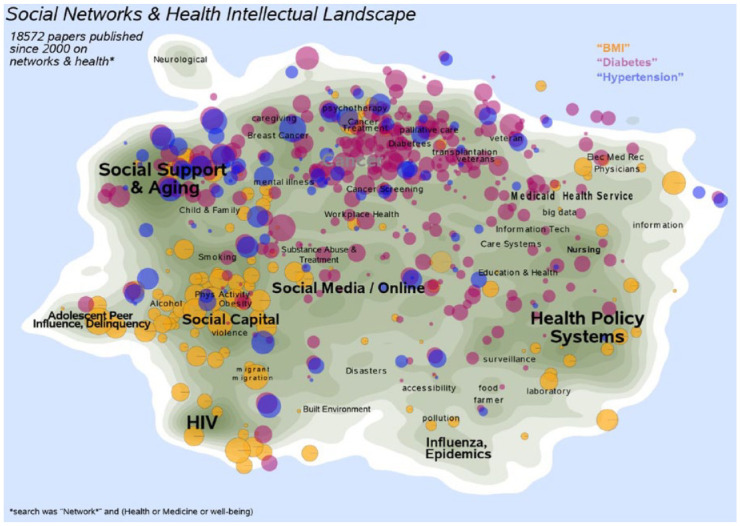
Body Mass Index (BMI), Diabetes, and Hypertension across Networks and Health Research Since 2000. *Note*: This figure shows the intellectual landscape of social networks and health research, based on modeling of English language articles indexed in Web of Science Social Science Citation Index on ((“health” or “well-being” or “medicine”) and “network”). There have been 18,572 such papers since 2000. Markers indicate articles with “BMI,” “diabetes,” or “hypertension” in the abstract. Points are sized proportionate to number of times cited.

To assess the literature’s current state, we use a text-network approach and visualize the key resulting topics (for details, “Appendix A: Analyses Details Regarding Main Text Figure 4 BMI, Diabetes, and Hypertension” in the online version of the journal). Overall, our results indicate that the most prominent areas of research on social networks and health are HIV, social capital, social support and aging, health policy systems, influenza and epidemics, social media or online, cancer, adolescent peer influence, and Medicaid. Notably, developmental stages (older adulthood, adolescence) seem to cluster with core topics most often researched alongside them.

Articles are rarely about one subject, and many topics overlap, but such overlap does not preclude related medical conditions from appearing in distinct parts of the social networks and health literature. Figure 4 shows where three key health topics—BMI, diabetes, and hypertension—fall across this intellectual landscape. We chose these topics to highlight three related health conditions that tend to co-occur with people who are obese, having adjusted odds ratios of 4.6 and 5.1, respectively, for hypertension and diabetes (Nguyen et al. 2008). Nevertheless, these terms fit in different areas of the social networks and health intellectual landscape (in Figure 4, proximity is important, but orientation is irrelevant). BMI research clusters in the lower middle with scholarship on adolescents, social support, and, to a lesser extent, HIV. Hypertension, in contrast, is prominent in the upper left, particularly centered on the clusters associated with aging and social support. Most of the overlap between these three terms is in research on social support and aging. These results highlight the sometimes particularistic nature of the social networks and health literature, where attempts to understand social processes rather than biomedical relations between comorbidities often drive the focus. At the same time, these results also highlight that further integration with the biomedical literature might push social networks and health researchers to consider more deeply how interrelated health conditions interact with network structures.

**Figure 5.**
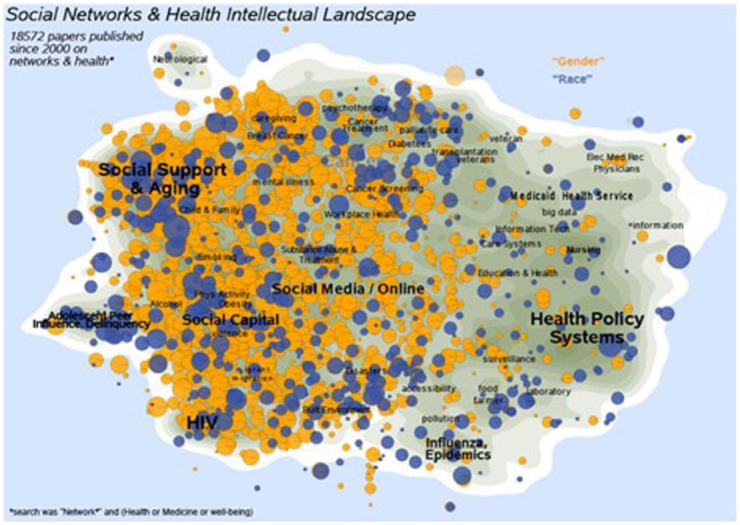
Race and Gender across Networks and Health Research Since 2000. *Note*: This figure shows the intellectual landscape of social networks and health research, based on modeling of English language articles indexed in Web of Science Social Science Citation Index on: ((“health” or “well-being” or “medicine”) and “network”). There have been 18,572 such articles since 2000. Points indicate articles with “race” or “gender” in the abstract. Points are sized proportionate to number of times cited.

One can also examine how subpopulations are studied across this landscape. Figure 5 displays articles that mention either “race” or “gender” in the abstract (point size proportional to times cited). Both race and gender are studied with other topics and thus found across the landscape. Race is more evenly distributed than gender, which is comparatively neglected in the more institutional topics represented on the left of Figure 5.

**Figure 6.**
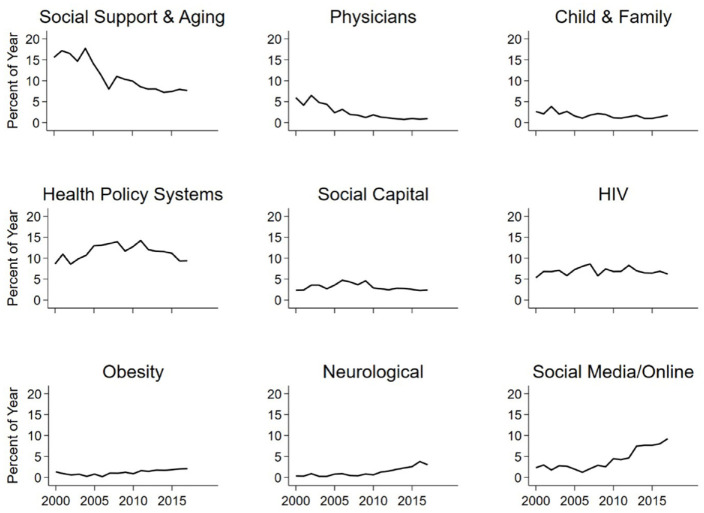
Publication Volume Trends in Social Networks and Health Since 2000. *Note*: This figure shows the percentage of articles published in each of the nine largest substantive areas defined via the clusters in Figures 4 and 5 in each year, allowing us to track the growth and decline of core topics in social networks and health. Within each year, the sum of all topics add to 100%, although these nine largest topics overall do not; smaller topics comprise the remainder.

While research on social networks and health continues to grow, it is unequal. Figure 6 reveals which topics have contributed to publication volume in this area since 2000 by graphing time trends in percentage of publication volume for each topic. Social support and aging dominates early—nearly 15% of publications—and online and social media or neurological-based research is rare. By 2020, social support and aging had fallen dramatically, whereas neurological and online/social media research have seen substantial growth.

## Future Directions

The past 20 years have uncovered challenges that point the way for major progress in the future of research on social networks and health, both in medical sociology and beyond. Recent work reveals, somewhat surprisingly, that name generator–based social network data collection can be a critical stumbling block. Decades of efforts have refined network data collection, most notably for egocentric approaches in the General Social Survey (GSS; Burt 1984), but research on increased social isolation in the 2004 GSS (McPherson, Smith-Lovin, and Brashears 2006) was hotly debated (Fischer 2009, 2011; McPherson et al. 2009) and ultimately deemed an artifact of poor network data collection practices (Paik and Sanchagrin 2013). This debate should continue to spur a deep look at ways to improve the validity of collected network data.

The prominence of the debate over social isolation called into question some classic means of network data collection, spawning new quantitative (adams 2019) and qualitative (Small 2017) treatises. The ability to collect such data well is important for understanding people’s social embeddedness through egocentric study designs (e.g., Perry and Pescosolido 2012) and because new tools allow sociocentric inference from such samples (Krivitsky and Morris 2017; Smith 2012). Furthermore, the continued formalization of qualitative network approaches (Hollstein 2011), especially as applied to health (e.g., Small 2017), has highlighted mechanisms through which networks can influence health that traditional survey-based approaches often miss (e.g., the avoidance of strong ties during help-seeking). Such approaches increasingly dovetail with more technologically sophisticated means of network data collection, including through cell phones, wearables, websites, and other sources (Eagle, Pentland, and Lazer 2009; Young, Fujimoto, and Schneider 2018), which permit *partially* sociocentric network measurement (Handcock and Gile 2010; Khanna et al. 2018; Mouw and Verdery 2012).

### Future Directions: The Role of Technology and Data

As technology continues to advance, and the capture of digital trace data through myriad wearable devices and smartphone applications practically tracks location data, calories burned, sleeping hours, and so forth, health data will become more prevalent and more easily linked to various measures of social networks. Already researchers are beginning to estimate physical activity by gender across space using mobile phones (Cesare et al. 2019). Intermittent survey cycles are one of the greatest challenges in health and social sciences, but technological advances are enabling unique, constantly captured network designs (Aral and Nicolaides 2017). New work also experiments with online communities, allowing novel insights into how network structure might influence health behavior spread (Centola 2010); opportunities in this space are only growing (Shirado and Christakis 2017). Technological adoption and advances will herald new and promising ways to measure where people go, what they do, and who they interact with, enabling new insights into the relationship between social networks and health. However, with these opportunities come challenges, especially as relates to core ethical considerations: respect for persons, beneficence, and justice. Two areas of notable concern are privacy and consent.

Evolving communication modalities will also affect social networks and health research. This fact has been crystalized with recent travel restrictions due to the COVID-19 pandemic. Changing how people communicate changes their social ties, and this implicates health (Sayers and Rhoades 2018). Data collection must keep up with the times—perhaps through video mining for expression and pitch. Communication in new ways may be challenging and stressful for some and comforting for others; new health concerns might arise (Ihm 2018).

New sources of administrative data promise major advances for social networks and health. For instance, the Longitudinal Employer Household Dynamics program (Abowd, Haltiwanger, and Lane 2004) contains data linking taxpayers to employers (which can be turned into longitudinal networks), and linked representative census samples (e.g., https://usa.ipums.org/usa/linked_data_samples.shtml) enable studying long-run kinship networks. Data from electronic medical records, like Medicare, allow researchers to examine patient-sharing (Perry et al. 2019), comorbidity (Iwashyna et al. 2009), and intrahousehold contagion (de Vaan and Stuart 2019). Death record linkages (e.g., Muennig et al. 2011) create new opportunities for understanding social network contributions to long-term health outcomes.

### Future Directions: A COVID-19 Silver Lining

Finally, COVID-19 may act similarly to the HIV epidemic. The number of articles referring to COVID-19 grows daily (adams, Light, and Theis 2020), and although most deal with pharmacological research from drug trials or updates about the virus itself, many are related to models and techniques aimed at identifying the spread and costs of the disease. Failed predictions from classic models have left many feeling misled by science, and new approaches will be needed. Governments around the world have expedited funding and grants toward projects related to COVID-19. The situation mirrors that of HIV, although much faster, and may lead to new innovations in social networks and health research—after all, the virus spreads through interpersonal contact, and there are numerous questions about the association between mitigation efforts and social isolation in the Internet era.

### Future Directions: Reaching Backward and New-Old Theoretical Avenues

Most research on social networks and health embraces “connectionist” theories, where the primary concern is with how health-related resources, ideas, or behavior diffuse through a network. There is an older sociological tradition in the study of networks that examines how social exchange patterns on multirelational networks reflect deep social institutions and norms (Bott 1971; Nadel 1957). This approach captures how overlapping patterns of relations entail rights and obligations with respect to others and how such informal roles emerge from interaction, allowing examination of the intertwining of social relations and identity (e.g., Mueller and Abrutyn 2016) and the emergence of communal norms (Molm, Collett, and Schaefer 2007). It is a growing area: Recent work connects the deaths of despair literature to social network considerations (Pescosolido, Lee, and Kafadar 2020) and advances Durkheimian theory (Mueller et al. 2021). Although not tied explicitly to health, a growing share of this research emphasizes the overlap, or not, of value systems among various subpopulations (DellaPosta 2020; DellaPosta, Shi, and Macy 2015). At the population level, simulation-based analyses are promising (e.g., Pescosolido et al. 2020; Verdery 2015; Verdery et al. 2020; Verdery and Margolis 2017). We are hopeful that this rich body of structural research continues to expand in future work.

## Conclusion

Recent decades have seen growing acceptance of the social determinants of health framework (Marmot and Wilkinson 2006). A key social determinant is the set of people one interacts with, through friends, family, and other networks. As such, understanding the links between social networks and health is vital for explicating the underlying mechanisms driving social determinants of health. Wider social trends promise to further expand the scope of network data available for researchers and test theories with more fine-grained detail than ever before.

The growth in this literature has been robust for the last 20 years, with no signs of abatement. In almost Tardis-like fashion, each door scholars open seems to bring a room full of even more questions to attend to. It is an open field (Light and Moody 2020). For example, understandings of the base social influence mechanisms are still largely rooted in a consensus-discussion psychological paradigm first elaborated over 60 years ago (Festinger, Schachter, and Back 1950). When do people form health-relevant beliefs? What characteristics of a relationship promote influence? Do the influences that shape health-appropriate behavior mirror those that shape risk, or do they act simultaneously? Does the stress induced by bridging social worlds create tension that leads to health risk (Bearman and Moody 2004), or does it provide access to diverse resources that promote health (Kamphuis et al. 2019)? What is the life course of a friendship, and how does one’s movement through that life course affect long-term health outcomes? How do network dynamics funnel flow of disease through a population? These and other questions remain unanswered. Sociologists, including medical sociologists, initiated the exponential growth on networks and health, and they are uniquely positioned to answer such questions in the coming decades.

## Supplemental Material

sj-docx-1-hsb-10.1177_00221465221086532 – Supplemental material for Analytic Advances in Social Networks and Health in the Twenty-First Century Click here for additional data file. Supplemental material, sj-docx-1-hsb-10.1177_00221465221086532 for Analytic Advances in Social Networks and Health in the Twenty-First Century by Alexander Chapman, Ashton M. Verdery and James Moody in Journal of Health and Social Behavior

## Notes

1 Research on graph theoretic problems in social systems predates the application of such ideas to interpersonal contacts (e.g., Euler 1741).

2 Google Scholar has higher citation counts than other sources (e.g., Web of Science or Scopus) because it indexes dissertations, reports, books, and non-English items with superior coverage, although counts from these three sources are highly correlated across fields (Martín-Martín et al. 2018). An analysis of all articles published in a sociological journal found that the linear relationship between Google Scholar (GS) and Web of Science (WoS) citation counts was GS = 24.84 + 1.976 × WoS in that field (Alwin and Verdery 2019).

3 Our index sources are likely incomplete in two ways (a) some items may be omitted—certain journals, almost all books, and so on—and (b) nuanced meanings of “health” or “networks” that could be included or excluded in our keyword-based searches. These biases likely lead to undercounts of total production. However, there are no reasons to expect such biases to change trends significantly.

4 There are two streams of social capital research; one follows the work of Bourdieu (1986), and the other stream follows Putnam (Carpiano 2006).

5 “Add Health” was originally named the “Teen Sex” study and paired with what later became the National Social Life, Health, and Aging Project—with the University of North Carolina focusing on adolescents and Chicago on older adults. That instantiation met with strong congressional opposition. But the crux of the study, including the focus on adolescent embeddedness in multiple social contexts, was saved by the strong advocacy of key National Institutes of Health personnel, such as Christine Bachrach. The Add Health network design owes to early work by Coleman and Hollingshead—with the focus on peers and crowds as a core social determinant of health and the proximate work of Robert and Beverley Cairns (Cairns et al. 1995) and Ennet and Bauman (1994) on smoking behavior.

6 Looking at all articles in journals WoS tags as sociology, from 1975 to 1999, we found 85 articles on networks and health out of 61,157 sociological articles (.139%). Since 2000, we found 1,091 networks and health articles out of 143,696 sociological articles (.759%).
