# Supplementary material for: Analytic Advances in Social Networks and Health in the Twenty-First Century
Source: J Health Soc Behav. 2022 Apr 8;63(2):191–209. doi: 10.1177/00221465221086532 (PMC9149133; doi:10.1177/00221465221086532)
Supplement: sj-docx-1-hsb-10.1177_00221465221086532 – Supplemental material for Analytic Advances in Social Networks and Health in the Twenty-First Century [file sj-docx-1-hsb-10.1177_00221465221086532.docx]

**Journal** of **Health**

and **Social Behavior**

OFFICIAL JOURNAL OF THE AMERICAN SOCIOLOGICAL ASSOCIATION

**ONLINE SUPPLEMENT**

**to article in**

Journal of Health and Social Behavior

**Analytic Advances in Social Networks and Health in the Twenty-First Century**

**Alexander Chapman**

*The Pennsylvania State University*

**Ashton M. Verdery**

*The Pennsylvania State University*

**James Moody**

*Duke University*

List of Appendices

*Supplemental Online Appendix A: Data Collection and Analyses, Main Text Figures 2, 3, and 4*

Analyses details for Figure 2 Social science publications

Analyses details for Figure 3 Publication volume and citations

Analyses details for Figure 4 BMI, diabetes, and hypertension

*Supplemental Online Appendix B: Trends in citation counts by publication cohort analyses*

Figure B1: Trends in citation counts to social networks and health research in sociology journals, by journal grouping and publication cohort

Analyses details regarding Figure B1 trends in citation counts

Appendix A: Analyses details for Figure 2 Social science publications

On the X-axis of Figure 2 (see main text) is publication year and on the Y-axis is the number of new publications. We graph articles indexed in Web of Science Social Science Citation Index using the search terms (*"health"* or *"well being"* or *"medicine"*) and *"network*".*

Appendix A: Analyses details for Figure 3 Publication volume and citations

In Figure 3 (see main text), we show publication volume and citations in articles categorized as sociological by Web of Science since 1975 on the same search terms that we used for Figure 2 (see main text and Appendix A: Analyses details for Figure 2). Figure 3 contains two panels. On the left, we graph the cumulative number of publications from three separate categories of journals, *JHSB* alone, the combination of *American Sociological Review*, *American Journal of Sociology*, and *Social Forces* (“Top Soc”), and the final grouping encompasses all other journals inclusive of about 225 total outlets (“Other Soc”). On the right, we plot the cumulative number of citations of those publications using the same categories. We cap counts in the other journal category to maintain the scale of our figure.

Appendix A: Analyses details regarding main text Figure 4 BMI, diabetes, and hypertension

To assess the literature on social networks and health, we use a text-network approach to identify topics (see Figure 4 in the main text). Topics are based on co-word models initially developed in bibliometrics (He 1999; Law et al. 1988) and used in prior work on sociology (Moody and Light 2006) and wider science studies (Edelmann, Moody, and Light 2017). We elaborate standard co-word models with modern language-aware text-parsing tools (SAS 2015). The intuition behind these models is that documents are similar to the extent that they share differentiating terms, creating a network of documents linked by terms, with comparatively rare terms counting more heavily than very common terms.

Once constructed, we cluster the paper-similarity network using a well-vetted network clustering technique (Blondel et al. 2008). We then review each cluster by hand to ensure (a) internal consistency and (b) external differentiation, splitting or merging clusters respectively if they are inconsistent. We review the most highly-weighted terms found in each cluster and use that to identify a name. Once clustered, we then visualize the network using the Fruchterman-Reingold network layout algorithm (Fruchterman and Reingold 1991), a technique that places papers that share many terms nearby. Overall orientation (up/down; right/left) is irrelevant in such maps; proximity is the core idea. As large, dense networks are difficult to visualize, we abstract to a 2-dimensional kernel density to identify regions in the space that have larger numbers of papers. Text labels for each topic are located at the median x-y coordinate of that cluster, sized according to frequency of papers in that cluster.

Appendix B: Figure B1 Trends in citation counts by cohort

**Figure B1.** **Trends in citation counts to social networks and health research in sociology journals, by journal grouping and publication cohort.**


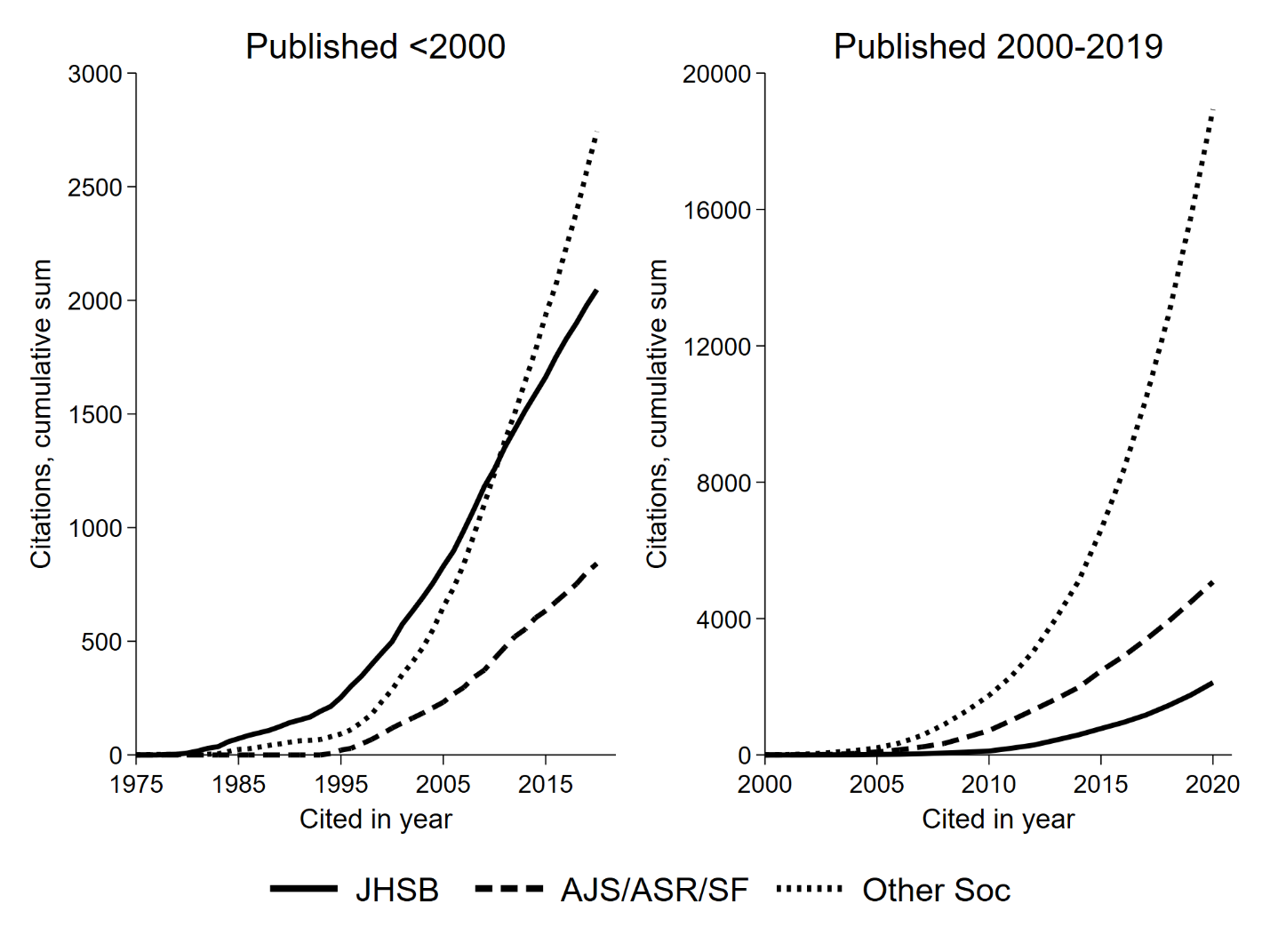


*Notes. This figure shows the cumulative number of citations to social networks and health articles per year according to Web of Science for articles published in three categories of sociology journals: Journal of Health and Social Behavior (JHSB); American Journal of Sociology, American Sociological Review, or Social Forces (AJS/ASR/SF); or all other journals* *Web of Science indexes in its sociology category (Other Soc). The left panel focuses on articles published prior to 2000, and shows citation counts from 1975 to present. The right panel shows articles published after 2000, with citations counts from then to present. The distinction between the citation counts in this figure and Figure 2 is that this figure shows numbers of citations made in each year to articles in those journals published in the delineated window (e.g., by 2020 there had been approximately 2,000 citations to social networks and health articles published in JHSB before the year 2000), whereas Figure 2 shows cumulative numbers of citations by a given year to articles ever published in that journal.*

Appendix B: Details regarding Figure B1

In Figure B1, we plot similar information to that of Figure 3 in the main text, except we plot the cumulative citations of articles published before 2000 on the left and after 2000 on the right by journal group. The clear trendline here is that *JHSB* led the breakthrough in terms of highly influential sociological research on networks and health through 2005. After 2000 JHSB’s dominant influence appears to be waning somewhat compared to research published in the scores of other sociology journals, though we note that *JHSB* articles have still garnered a great share with some 2,000 citations in the 20-year span from 2000-2020 compared with close to 5,000 combined among *ASR*, *AJS*, and *Social Forces* and nearly 20,000 for the remaining sociology outlets.

**References**

Blondel, Vincent D., Jean-Loup Guillaume, Renaud Lambiotte, and Etienne Lefebvre. 2008. “Fast Unfolding of Communities in Large Networks.” *Journal of Statistical Mechanics: Theory and Experiment* 10.

Edelmann, Achim, James Moody, and Ryan Light. 2017. “Disparate Foundations of Scientists’ Policy Positions on Contentious Biomedical Research.” *Proceedings of the National Academy of Sciences* 114(24):6262–67.

Fruchterman, Thomas MJ, and Edward M. Reingold. 1991. “Graph Drawing by Force-Directed Placement.” *Software: Practice and Experience* 21(11):1129–64.

He, Qin. 1999. “Knowledge Discovery Through Co-Word Analysis.” *Library Trends* 48(1):133–59.

Law, John, Serge Bauin, J. Courtial, and John Whittaker. 1988. “Policy and the Mapping of Scientific Change: A Co-Word Analysis of Research into Environmental Acidification.” *Scientometrics* 14(3–4):251–64.

Moody, Jim, and Ryan Light. 2006. “A View from above: The Evolving Sociological Landscape.” *The American Sociologist* 37(2):67–86.

SAS. 2015. *Base SAS 9.4 Procedures Guide*. SAS Institute.
